# Surgical reconstruction of coexisting left ventricular aneurysm and pseudoaneurysm in a patient with ventricular tachycardia: a case report

**DOI:** 10.1093/ehjcr/ytag116

**Published:** 2026-02-14

**Authors:** María Belén Solís Chávez, Manuel Carnero Alcázar, Pedro Marcos-Alberca Moreno, María Vidal Martínez, Luis Carlos Maroto Castellanos

**Affiliations:** Department of Cardiac Surgery, Hospital Clínico San Carlos, C/ Professor Martín Lagos s/n, 28040 Madrid, Spain; Department of Cardiac Surgery, Hospital Clínico San Carlos, C/ Professor Martín Lagos s/n, 28040 Madrid, Spain; Department of Cardiovascular Imaging, Hospital Clínico San Carlos, C/ Professor Martín Lagos s/n, 28040 Madrid, Spain; Radiology Department, Hospital Clínico San Carlos, C/ Professor Martín Lagos s/n, 28040 Madrid, Spain; Department of Cardiac Surgery, Hospital Clínico San Carlos, C/ Professor Martín Lagos s/n, 28040 Madrid, Spain

**Keywords:** Case report, Ventricular tachycardia, Left ventricle, Myocardial infarction, Cardiac magnetic resonance, Computed tomography, Coronary angiography

## Abstract

**Background:**

Left ventricular (LV) aneurysm and pseudoaneurysm are rare but potentially life-threatening complications of myocardial infarction. Their coexistence poses significant diagnostic and surgical challenges.

**Case summary:**

A 69-year-old woman with type II diabetes, hypertension, and dyslipidaemia presented with sustained ventricular tachycardia while remaining haemodynamically stable. Multimodality imaging revealed a chronic myocardial infarction with complete right coronary artery occlusion, a large LV aneurysm, and a focal pseudoaneurysm. Urgent LV reconstruction using a Dacron patch was performed. The postoperative course was uneventful, with preserved sinus rhythm and improved ventricular function.

**Discussion:**

This case underscores the importance of comprehensive imaging to characterize complex post-infarction LV remodelling. Surgical exclusion of scarred myocardium effectively restored LV geometry, improved cardiac function, and reduced arrhythmic risk. The successful repair provides insight into the decision-making process regarding optimal timing and technique for LV reconstruction.

Learning points

- LV aneurysm can result from silent transmural myocardial infarction.
- Recurrent ventricular arrhythmias are an indication for LV surgical repair.

## Introduction

Left ventricular (LV) aneurysm is a recognized complication of transmural myocardial infarction, caused by infarct-related myocardial thinning and maladaptive remodelling. LV pseudoaneurysm, in contrast, results from myocardial rupture contained by the pericardium or scar tissue, and lacks myocardial layers in its wall, carrying a high risk of rupture.^1^

The coexistence of a true LV aneurysm and pseudoaneurysm is exceedingly rare, with only isolated cases reported. One prior case described such coexistence in the context of a silent inferior infarction.^2^ Beyond heart failure progression, both lesions predispose to malignant ventricular arrhythmias arising from the scar border zone. Surgical LV reconstruction can restore ventricular geometry, improve LV function, and may eliminate arrhythmogenic substrate.^3^

We report a 69-year-old woman presenting with sustained ventricular tachycardia who was ultimately diagnosed with both an LV aneurysm and a pseudoaneurysm. This case illustrates the importance of multimodality imaging and timely surgery in this complex presentation.

## Summary figure

**Figure:**
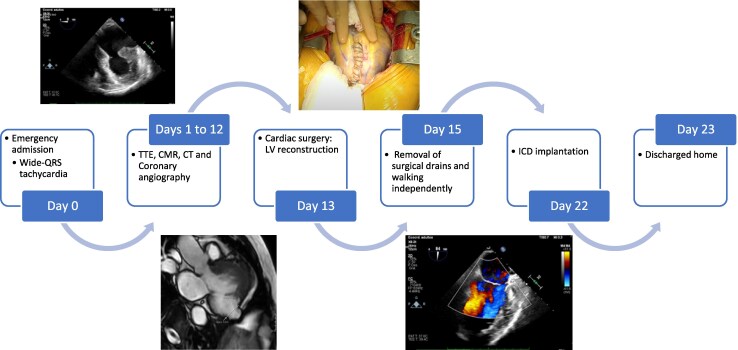
Summary timeline showing the clinical course from emergency admission to discharge. CMR: Cardiac Magnetic Resonance, CT: Computed Tomography, ICD: implantable cardioverter defibrillator, TTE: transthoracic echocardiogram.

## Case presentation

A 69-year-old woman presented with palpitations. The ECG revealed a regular wide-QRS tachycardia at 150 bpm with right bundle branch block (RBBB) morphology and a superior axis. She remained haemodynamically stable. Her medical history included hypertension and dyslipidaemia, with no prior hospital admissions.

The most likely diagnosis was ventricular tachycardia, and a procainamide bolus (100 mg IV) was administered. Cardioversion to sinus rhythm was achieved, revealing Q waves in the inferior leads. The chest X-ray was normal. Initially, antiarrhythmic treatment was withheld due to the absence of recurrences, but a low-dose beta-blocker (bisoprolol 1.25 mg/24 h) was later initiated.

**Figure 1:**
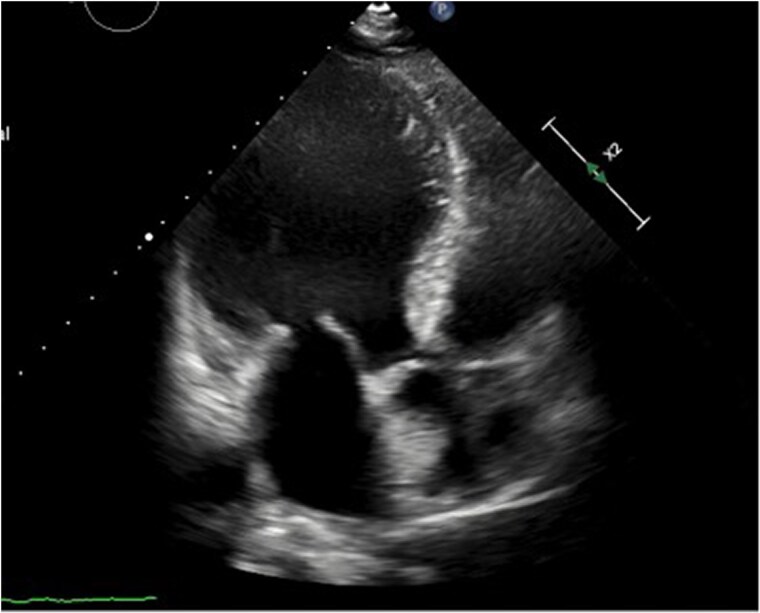
Pre-operative transthoracic echocardiogram. Three-chamber apical view showing a basal left ventricular aneurysm with thinned, dyskinetic wall segments.

Echocardiography demonstrated a mildly dilated LV with a thinned inferolateral wall and basal-to-mid aneurysmal sac segments (*Figure 1*). The left ventricular ejection fraction (LVEF) was moderately reduced at 40%. Right ventricular function was preserved. No significant valvular disease was detected.

**Figure 2:**
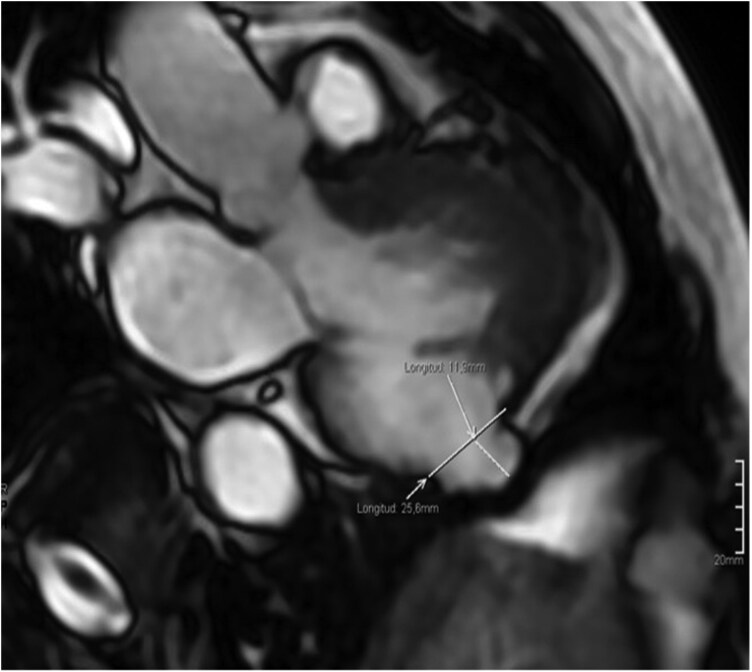
Cardiac MRI. Three-chamber view SSFP (Steady-State Free Precession) sequences or Cine. A large aneurysm in the basal and mid-inferolateral segments of the left ventricle with a saccular pseudoaneurysm (11 × 21 mm) at the aneurysm border.

Cardiac magnetic resonance (CMR) revealed marked LV dilatation, an aneurysm involving the basal and middle segments of the inferior and inferolateral walls, with markedly thinned myocardium, and a small saccular deformity (11 × 21 mm) above the aneurysm, consistent with pseudoaneurysm (*Figure 2*). The LVEF was 29%. Gadolinium-enhanced imaging showed transmural ischaemic scarring in all thinned segments. The findings were consistent with a chronic myocardial infarction in the right coronary artery territory, with unfavourable remodelling, severe dysfunction, and a focal pseudoaneurysm.

**Figure 3:**
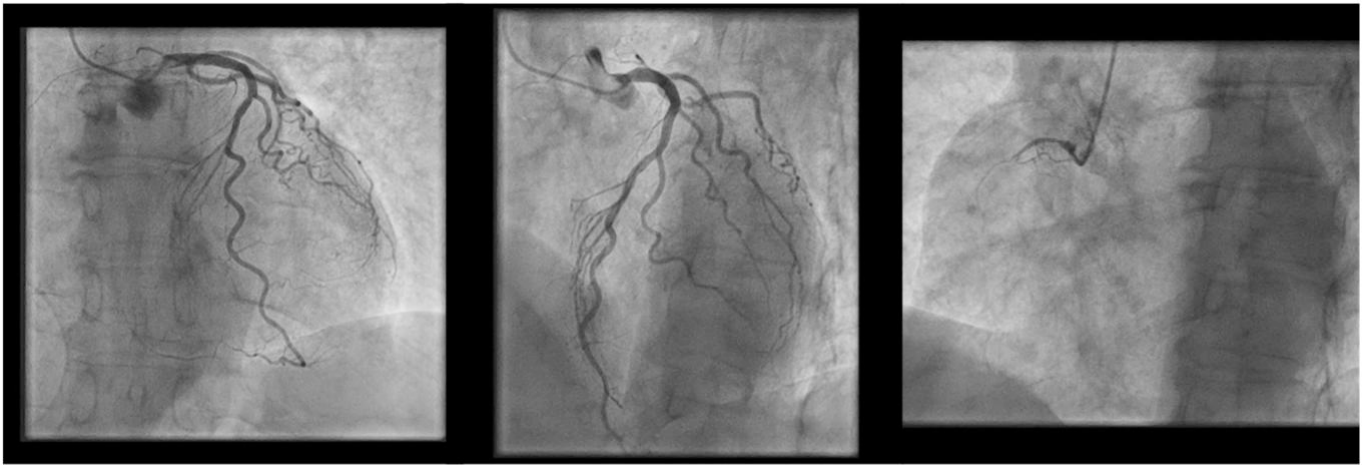
Coronary Angiographic findings. (*A*) LAO caudal view showing LMA, LAD, and LCX with no stenosis. (*B*) Cranial view of the left coronary system confirming unobstructed flow. (*C*) LAO caudal view of RCA showing proximal chronic total occlusion, with retrograde filling via collaterals. LAO: left anterior oblique.

Coronary angiography demonstrated chronic proximal right coronary artery (RCA) occlusion with collateral supply from the left system; the remaining vessels were unobstructed (*Figure 3*).

CT coronary angiography confirmed chronic ischaemia with complete RCA occlusion and distal perfusion through collateral circulation. Additionally, aneurysmal dilation in the basal inferolateral LV wall and a pseudoaneurysm were observed. Given these findings, urgent surgical repair was indicated.

**Figure 4:**
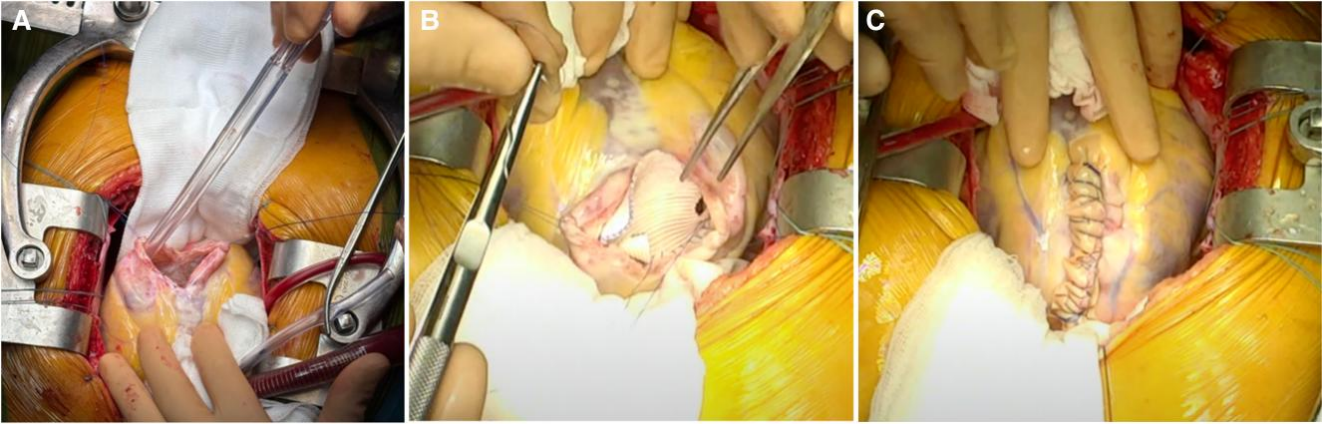
Surgical procedure of left ventricular aneurysm and pseudoaneurysm repair. (*A*) Opening of the inferior left ventricular wall and placement of stay sutures to keep the pseudoaneurysm open. (*B*) Suturing the Dacron patch to the ventricular wall rim of the aneurysm. (*C*) Closure and reinforcement of the new apex using two pericardial patches to strengthen and repair the LV pseudoaneurysm walls.

The surgical approach was performed through a median sternotomy with cardiopulmonary bypass (CPB), given that the lesions were located in the inferior and apical LV walls, with free-wall rupture contained in the inferolateral segment. The aneurysm rim was first identified and excluded using a Dacron patch sutured with a 3-0 polypropylene suture from the mitral annulus to the base of the papillary muscles (*Figure 4C*). No organized thrombus was found within the LV cavity. The ventriculotomy was then reconstructed using a continuous suture supported by two pericardial (Edwards bovine pericardial patch) strips in a sandwich fashion (*Figure 4D*). Transthoracic echocardiography (TTE) confirmed complete exclusion of the aneurysmal cavity without residual flow (see Supplementary material online, *video S2*).

**Figure 5:**
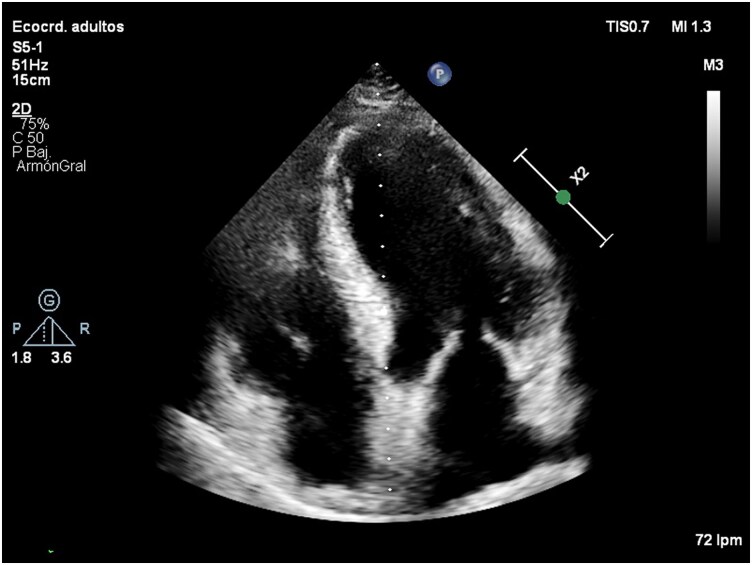
Follow-up transthoracic echocardiogram after surgical repair. Four-chamber apical view. Improved global systolic function with LVEF of 58%.

The patient had an uneventful postoperative course without rhythm disturbances. A follow-up echocardiogram showed concentric LV remodelling, improved LVEF (58%), type II diastolic dysfunction, and only mild mitral regurgitation (*Figure 5*).

During hospitalization, the Heart Team recommended placement of an implantable cardioverter-defibrillator (ICD) as secondary prevention. She was discharged 24 h after ICD implantation on postoperative day 8.

## Discussion

Left ventricular aneurysm is defined by akinetic or dyskinetic motion of the LV wall, often associated with a reduced ejection fraction. True LV aneurysms typically develop after transmural infarction through progressive wall thinning and adverse remodelling.^1^ Indications for surgical ventricular restoration include LV aneurysm in combination with symptoms such as angina, congestive heart failure, or ventricular tachycardia.^4^ One of the most common surgical techniques for LV aneurysm repair is the Dor procedure.^5^

Left ventricular pseudoaneurysm is an infrequent, yet severe and potentially lethal mechanical complication of previous myocardial infarction, resulting from LV free-wall rupture contained by adjacent pericardium^1^ . Acute LV pseudoaneurysms are a direct indication for urgent surgery, even in asymptomatic patients, due to the high risk of rupture. However, in subacute or chronic LV pseudoaneurysm, the false cavity stabilizes, and the risk of rupture is significantly reduced. The surgical decision must consider anatomic features to allow effective reconstruction.

The clinical presentation of LV aneurysm and pseudoaneurysm varies widely and may include angina pectoris, dyspnoea, and heart failure, among others. The ECG often displays non-specific ST-segment alterations.^1^ Our patient had no history of known ST-elevation myocardial infarction but presented to the emergency department with a regular wide-QRS tachycardia. Ventricular arrhythmias commonly originate from the border zone as scar-related re-entrant arrhythmias.

Multimodality imaging is central to distinguishing LVP from a true aneurysm. Echo and CMR/CT suggest LVP when a narrow neck connects the LV cavity to a saccular outpouching, often with bidirectional colour-Doppler flow; CMR additionally demonstrates transmural scar and helps define the pseudoaneurysm wall composition. In contrast, true aneurysms have a broad neck and a wall of scarred myocardium continuous with the endocardium. In our patient, echocardiography, CMR, and CT jointly defined a large true aneurysm with an adjacent focal pseudoaneurysm.

Unlike an acute LV pseudoaneurysm, this case represents a subacute or chronic presentation, the timing of surgery may be subject to debate within the Heart Team. Considering the risk of rupture (primarily from the pseudoaneurysm), the patient’s haemodynamic stability with preserved functional status, and the risk of sudden cardiac death due to ventricular tachycardia, the decision was made to perform isolated LV reconstruction during hospitalization.

In our case, a Dacron patch was used to exclude the aneurysm endoventricularly (Dor procedure), thereby ensuring exclusion of the white fibrous scar tissue, avoiding friable myocardium under direct visualization and enabling suturing at the border zone (the transition between thinning walls and the normal myocardium). A well-established sandwich technique using two pericardial patches was also employed, a method successfully applied in various LV pseudoaneurysm repairs.^6^ This closure technique for the LV cavity allows haemostatic suture lines, potentially reducing the risk of bleeding.

Postoperatively, the patient maintained sinus rhythm, confirming the adequacy of border-zone suturing and reducing the likelihood of a scar-related re-entrant mechanism. Echocardiography showed significant improvement, demonstrating the effectiveness of the surgical procedure in restoring and reconstructing the LV. Although minimally invasive and hybrid techniques have been explored for LV reconstruction in selected patients, our case demonstrates that conventional open repair can still achieve excellent outcomes. Indeed, the patient experienced a rapid postoperative recovery, regaining the ability to walk within 48 h, underscoring the safety and effectiveness of traditional surgical reconstruction when performed in carefully selected patients.

## Patient’s perspective

The patient expressed surprise at being diagnosed with both an aneurysm and a pseudoaneurysm after presenting only with palpitations, but reported being satisfied with the surgical outcome and smooth recovery.

## Conclusion

This case underscores the importance of multimodality imaging in differentiating LV aneurysm and pseudoaneurysm, both rare but high-risk post-infarction complications. Surgical reconstruction can restore LV function, reduce arrhythmic risk and prevent rupture. Early recognition and timely intervention are key to favourable outcomes.

## Lead author biography


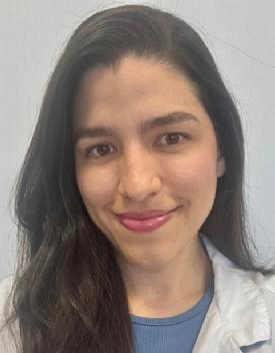


María Belén Solís Chávez is an Ecuadorian physician currently in her third year of residency in Cardiac Surgery at Hospital Clínico San Carlos in Madrid, Spain.

## Supplementary Material

ytag116_Supplementary_Data

## Data Availability

The data underlying this article cannot be shared publicly due to patient privacy constraints. De-identified data may be made available from the corresponding author upon reasonable request.

## Notes

**Consent:** The authors confirm that written consent for the submission and publication of this case report, including images and associated text, has been obtained from the patient in accordance with COPE guidance.
