# Supplementary figures and images for: Surgical reconstruction of coexisting left ventricular aneurysm and pseudoaneurysm in a patient with ventricular tachycardia: a case report
Source: Eur Heart J Case Rep. 2026 Feb 14;10(3):ytag116. doi: 10.1093/ehjcr/ytag116 (PMC12965326; doi:10.1093/ehjcr/ytag116)

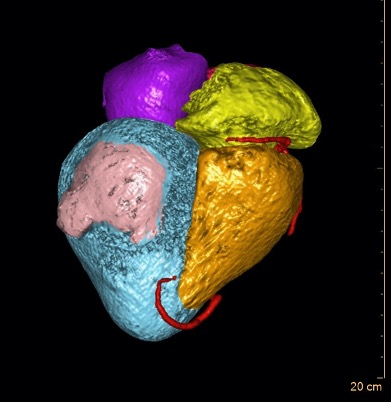

Supplement: ytag116_Supplementary_Data [file ytag116_supplementary_data.zip › Supplementary C. 3D CT with colors.JPG]

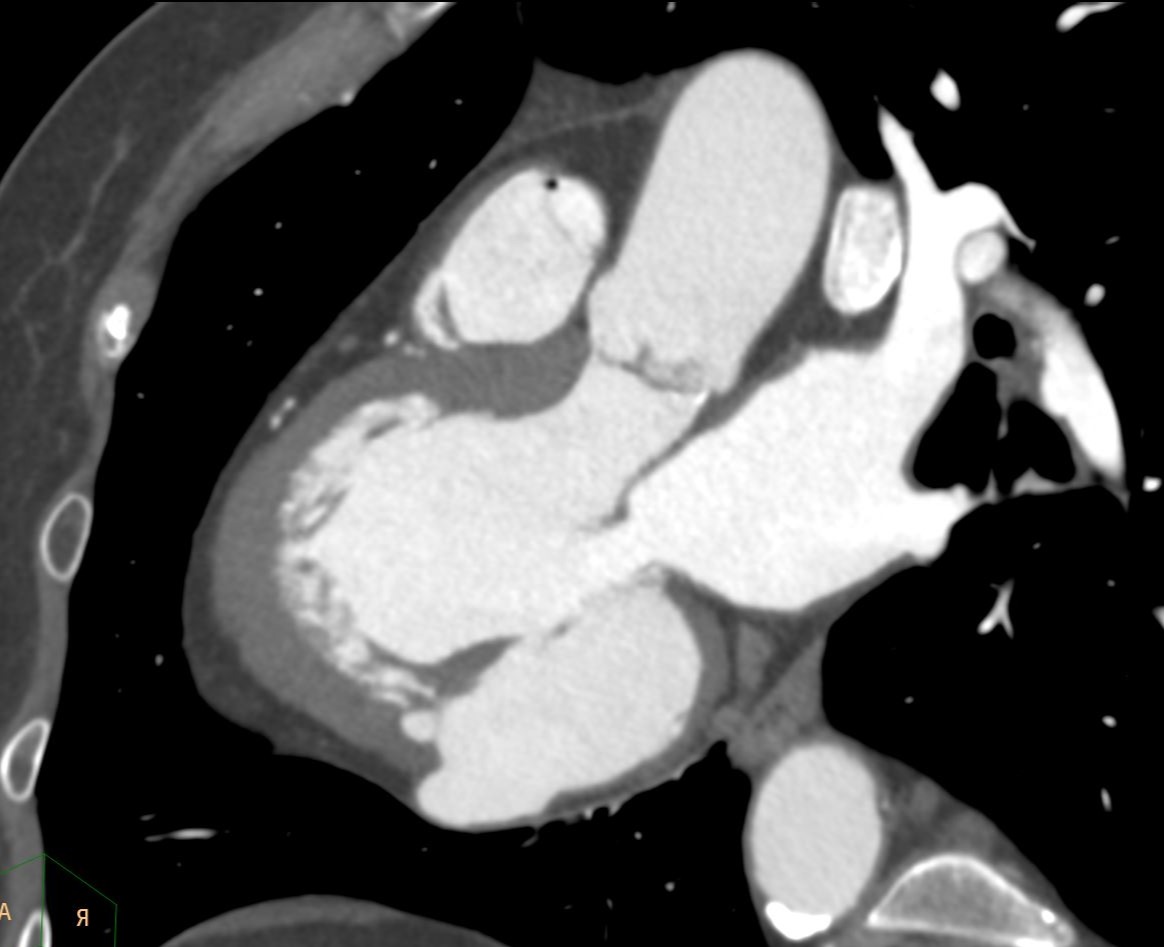

Supplement: ytag116_Supplementary_Data [file ytag116_supplementary_data.zip › Supplementary 1A. MPR PEL .JPG]

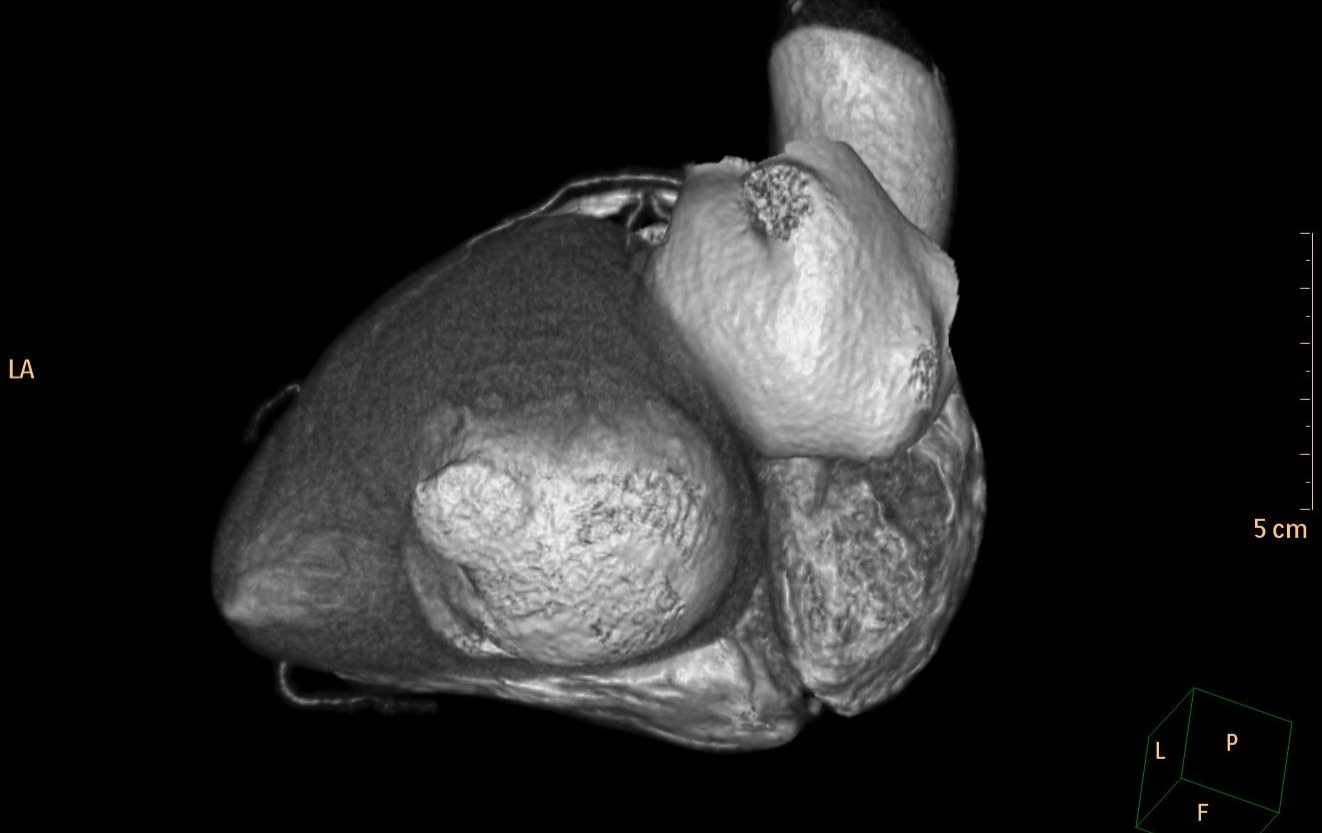

Supplement: ytag116_Supplementary_Data [file ytag116_supplementary_data.zip › Supplementary B. 3D CT.JPG]
